# The *VEGFA* rs3025039 Variant Is a Risk Factor for Breast Cancer in Mexican Women

**DOI:** 10.3390/ijms251810172

**Published:** 2024-09-22

**Authors:** Bricia M. Gutiérrez-Zepeda, Mariana M. Gómez-Del Toro, Diego J. Ortiz-Soto, Denisse S. Becerra-Loaiza, Angel F. Quiroz-Bolaños, Antonio Topete, Ramón A. Franco-Topete, Adrián Daneri-Navarro, Alicia Del Toro-Arreola, Antonio Quintero-Ramos

**Affiliations:** 1 Doctorado en Genética Humana, Departamento de Biología Molecular y Genómica, Centro Universitario de Ciencias de la Salud, Universidad de Guadalajara, Guadalajara 44340, Mexico; 2 Laboratorio de Inmunología, Departamento de Fisiología, Centro Universitario de Ciencias de la Salud, Universidad de Guadalajara, Guadalajara 44340, Mexico; 3 Licenciatura en Médico Cirujano y Partero, Centro Universitario de Ciencias de la Salud, Universidad de Guadalajara, Guadalajara 44340, Mexico; 4 Departamento de Aparatos y Sistemas II, Universidad Autónoma de Guadalajara, Zapopan 45129, Mexico; 5 Doctorado en Ciencias Biomédicas, Departamento de Fisiología, Centro Universitario de Ciencias de la Salud, Universidad de Guadalajara, Guadalajara 44340, Mexico; 6 Unidad de Investigación Biomédica 02, Hospital de Especialidades, Unidad Médica de Alta Especialidad, Centro Médico Nacional de Occidente, Instituto Mexicano del Seguro Social, Guadalajara 44340, Mexico

**Keywords:** VEGFA, breast cancer, rs3025039, Mexican woman, +936 C/T, SNV

## Abstract

Breast cancer (BC) is the leading cause of death from tumors in women worldwide, influenced by various factors, including genetics. The T allele of the single nucleotide variant (SNV) rs3025039 at position +936 of the *VEGFA* gene has been reported to affect the mRNA regulatory mechanisms, potentially altering *VEGFA* expression and increasing BC risk. This study aimed to investigate the association between rs3025039 and BC in Mexican women residing in Jalisco, Mexico. The study included 231 women with a confirmed diagnosis of BC and 201 healthy subjects as a reference group (RG). PCR–RFLP was employed for the genotyping of rs3025039, with the visualization of amplified products using polyacrylamide gel electrophoresis. Significant differences were observed in rs3025039 alleles and genotypes between BC cases and the RG (*p* = 0.0038). The frequency of the T allele and the CT genotype was higher in the BC group compared to the RG, with a significant difference (*p* = 0.0006). In conclusion, this research suggests that the SNV rs3025039 is associated with a higher risk of BC in Mexican women. These findings enhance our understanding of the genetic underpinnings of BC in this population, offering potential insights for future studies and interventions.

## 1. Introduction

Breast cancer (BC) is the most commonly diagnosed form of cancer, with the highest incidence, mortality, and prevalence worldwide, including among Mexican women [1]. Unfortunately, the mortality rate is increasing, highlighting the lack of resources for prevention, detection, and adequate treatment in low- and middle-income countries [2].

Several factors contribute to the development of BC, including reproductive, hormonal, lifestyle, ethnic, family history, environmental, and hereditary influences, among others [3]. Hereditary BC is specifically associated with mutations, principally in genes such as *BRCA1*, *BRCA2*, *PTEN*, *TP53*, *STK11*, *ATM*, and *NBS1* [4]. In addition, the vascular endothelial growth factor gene (*VEGFA*) [5], has also been linked to BC, since cancer cells require nutrients, oxygen, and the ability to eliminate waste products, where *VEGFA* is closely related. VEGF is a protein-encoding gene belonging to the growth factor family, which induces the proliferation and migration of vascular endothelial cells and the pathological angiogenesis [6]. Elevated plasma protein concentrations of *VEGFA* have been associated with an increased risk of BC development [7], and this increase can be due to amino acid change in some genetic variants present in the same gene.

**Figure 1:**
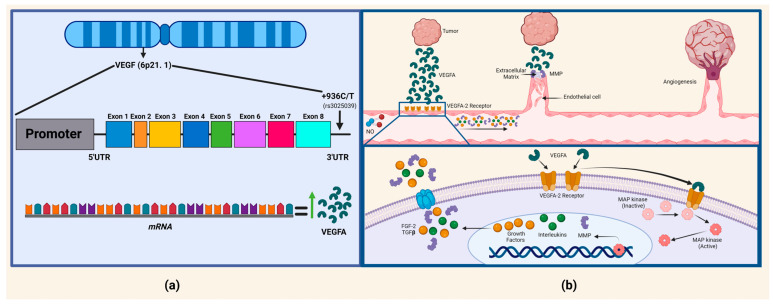
*VEGFA* gene locus and the pathophysiological mechanism of tumor angiogenesis. (**a**) The rs3025039 variant, located in the 3′UTR region of *VEGFA*, where the C allele has been associated with an increase in mRNA, consequently leading to elevated plasma VEGFA levels; however, the T allele results in the loss of a potential binding site for the transcription factor AP-4, leading to a reduction in the plasma levels of VEGFA. (**b**) The process of angiogenesis from pre-existing blood vessels can be observed. Initially, there is vasodilation in response to the presence of nitric oxide, causing an increase in vascular permeability due to the presence of VEGFA, growth factors, interleukins, and MMPs (matrix metalloproteinases). The basement membrane is degraded by MMPs, allowing for intracellular contact between endothelial cells through a plasminogen activator, facilitating their migration and proliferation. Finally, peritoneal cells are recruited, resulting in the formation of a mature blood vessel and thus producing angiogenesis [17].

The *VEFGA* gene is located at 6p21.3 and contains eight exons and seven introns. It presents a single nucleotide variant (SNV) rs3025039 at position +936, where there is a change from a C to a T allele. However, the rs3025039 variant may impact the regulatory mechanisms of mRNA generation in this region, leading to changes in gene expression (Figure 1) [8,9]. Additionally, the binding of hypoxia-induced proteins to VEGFA mRNA has been observed, substantially extending mRNA half-life. This post-transcriptional regulation impacts not only VEGFA but also other hypoxia-inducible genes, such as erythropoietin or tyrosine hydroxylase [10]. Elevated levels of tissue VEGFA are associated with an unfavorable prognosis and lower overall survival in patients with BC. However, carriers of +936 CT + TT *VEGFA* genotypes have a protective effect for BC as well [11].

It has been reported that the change from C to T in rs3025039 results in the loss of a potential binding site for the transcription factor AP-4 [12], which consequently leads to a reduction in the plasma levels of VEGFA. Consequently, carriers of the 936T allele have significantly lower VEGFA levels compared to non-carriers [13]. Furthermore, the T allele has been observed to be associated with a reduced uptake of 18F-fluorodeoxyglucose, which is used in the detection and staging of BC [11,12,13,14,15]. However, other studies have reported the opposite effect, suggesting that the C allele of the *VEGFA* +936 site is associated with an increased risk of BC in situ [16]. It is important to note that these findings are still inconclusive and further studies are needed to fully understand the relationship between rs3025039 and BC. Given the controversial role of VEGFA, we consider it important to evaluate the association of the *VEGFA* variant rs3025039 with BC risk in Mexican women.

## 2. Results

### 2.1. Description of Clinical Features

Clinical data acquired through patient questionnaires are presented in Table 1, revealing a predominantly postmenopausal population with a mean age of 55.3 ± 13.20. Many patients (31%) were classified with obesity based on their body mass index. Regarding pathological mechanisms, the prevalent molecular phenotype was luminal A (38%) and clinical stage II and III (86%), with 83% showing an absence of metastasis.

### 2.2. Genetic Association

**Figure 2:**
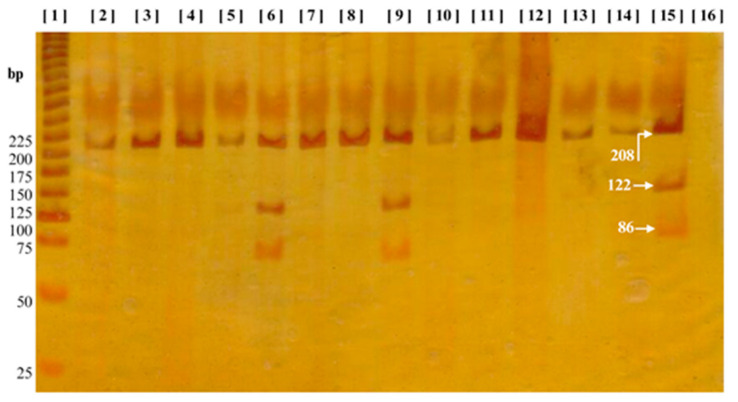
The electrophoresis results of the rs3025039 variant. In lane one, a molecular weight marker of 25 base pairs (bps) is depicted. Lanes 2–5, 7, 8, and 10–14 reveal a 208 bp band, representing the wild-type homozygous genotype C/C. Lines 6, 9, and 15 exhibit bands at 86, 122, and 208 bp, indicative of the heterozygous genotype C/T. Finally, line 16 shows the negative control.

The genotype distribution of rs3025039 in both groups, BC (*n* = 231) and the reference group (RG, *n* = 201), adhered to the Hardy–Weinberg equilibrium (*p* < 0.0001 and *p* = 0.41, respectively). A possible confounding variable in the study may lie in the reference group, as no clinical evaluations were performed to confirm their health status, specifically whether they were cancer-free. Information was obtained solely through a questionnaire and informed consent. Figure 2 displays the electrophoresis results for the rs3025039 variant, while Table 2 illustrates that allele C is predominant in both study groups. Notably, allele T exhibits a significant increase in the BC group compared to the RG (*p* = 0.0038). Furthermore, when both study groups were compared in the different inheritance models, co-dominant, dominant and overdominant, a significant difference was noted, with the presence of a heterozygous or homozygous genotype (C/T, T/T) prevailing with an increase in the BC group (*p* < 0.0001, Table 2).

BC patients were stratified according to their genotype and various clinical characteristics, including menopausal status, molecular phenotype, hormone receptors, vital status, and metastasis, as shown in Table 3.

However, the study exclusively identifies a significant relationship between the menopausal status of patients and their genotypes under the codominant inheritance model CC + CT (*p* = 0.0271).

## 3. Discussion

We consider it necessary to mention that the results shown in this study are specific to the Mexican population. These data should not be extrapolated to other populations due to the unique genetic structure of each group, at least in that they share genetic characteristics. It is important that similar studies be conducted in other regions of Mexico and around the world so they can be compared with the findings of this study in the near future. VEGFA is an important molecule that promotes angiogenesis and vasculogenesis, making it a risk factor for BC. This pathology exhibits significant clinical heterogeneity among patients, necessitating the use of different variables used to classify patients into various stages and to identify specific molecular markers to guide the selection of appropriate treatment [17]. In different populations, the luminal A molecular phenotype is the most frequent, with a reported frequency of 28% to 31% [17,18], which aligns with the analyzed population (38%). The proto-oncogene Her-2/neu (c-erb-B2) has gained growing significance as a prognostic and predictive factor in BC, with elevated expression or amplification linked to poorer outcomes among BC patients [19,20]; this is concerning because in the analyzed population, 22% of patients tested positive for this molecular marker. It is known that the earlier detection of BC, in stages I–II, can lead to a potential cure, whereas a more advanced stage of the disease produces a less favorable prognosis. Unfortunately, distant metastatic disease is currently beyond the reach of a cure [21,22], which means the majority of our patients were in clinical stage II, at 52%, which is treatable with a good prognosis, while only 17% of BC patients had metastasis.

Angiogenesis is a critical process in tumor growth, progression, and metastasis, involving the formation of new blood vessels [23]. The regulation of angiogenesis is intricate, and the *VEGFA* pathway plays a central role in this process [24]. While many angiogenic factors have been identified, *VEGFA* is considered the most crucial in promoting neovascularization and increased vascular permeability. This evidence suggests that angiogenesis precedes the transformation of mammary hyperplasia into malignant cancer. [25]. Studies have investigated the relationship between the rs3025039 variant in *VEGFA* and BC risk [26]. The rs3025039-C variant has been associated with slower disease progression compared to patients carrying the T allele. In Caucasian patients with the CT or TT genotype, the progression of BC was observed to be delayed by up to 3 months compared to those with the CC genotype, who experienced faster disease progression [27].

Angiogenesis, facilitated by VEGFA, is crucial for tumor growth and metastasis. The rs3025039 variant located in the 3′ untranslated region of the VEGFA gene has been shown to impact mRNA regulation, leading to altered VEGFA expression levels. Elevated VEGFA levels are associated with increased angiogenesis and consequently, a higher risk of cancer progression [10]. Renner et al. (2000) reported that a C to T change results in the loss of a potential binding site for the transcription factor AP-4. Individuals carrying the 936T allele exhibited significantly lower VEGFA plasma concentrations compared to non-carriers [10]. However, the study was conducted in healthy individuals, and in a tumor environment, the effect of the variant could be the opposite. Additionally, the T allele was associated with a reduced uptake of 18F-fluorodeoxyglucose, a substance used for detecting and staging BC [15].

In our study, we observed a higher prevalence of the C allele in patients with BC (69%) and the RG (78%) compared with the T allele, at 31% vs. 22%, respectively. Upon calculating the OR, we found a 1.61 [95% CI (1.18–2.19)] increased risk of developing BC in individuals carrying the T allele. In contrast to studies conducted in Iranian patients, no significant association was found between the variant frequency of *VEGFA* variants rs3025039, rs2010963, rs833061, and rs35569394 and BC risk or protection [28]; this is similar to findings in the Australian population, where the T allele was considered a protective factor for BC, with an OR of 0.51 [95% CI (0.38–0.70)] [29].

When comparing inheritance models, we found that the CT genotype of the codominant model showed a statistically significant association (*p* = 0.00001) with BC, with 60% of patients presenting this genotype compared to the RG. These results differ from a study by Rodrigues et al., which investigated a Spanish population and found a significant association (*p* = 0.046) with a recessive inheritance model (CT + TT) in patients aged 45–55 years. However, in their study, T allele carriers were associated with decreased BC risk, with *p* = 0.014; OR 0.67 [95% CI (0.48–0.92)] [11]. It is important to mention that although the sample size was reasonable, given the controversial results reported, we believe that larger genetic studies with bigger sample sizes are required in the future to confirm the findings. This would increase the statistical power of the study.

Throughout women’s reproductive years, which generally span from menarche to menopause, the ovaries produce steroid hormones that significantly impact breast development and function. It is well established that an early onset of menarche and a delayed onset of menopause increases the risk of BC in women [30]. Estrogens are a regulatory factor of angiogenesis; they increase angiogenesis by inducing the expression of VEGFA and the tie receptor (tyrosine kinase and EGF homologous domains), which promote endothelial survival and blood vessel stabilization [17]. However, when comparing clinical characteristics with the presence of the variants in BC patients, significance was only found postmenopause. It was observed that the heterozygous CT genotype produces a risk for BC, with an OR = 1.9, CI (1.0–3.38, *p* = 0.027).

This study supports the notion that genetic variations in *VEGFA* can significantly influence BC biology and patient outcomes.

The findings suggest that genotyping for the rs3025039 variant could be a valuable tool in assessing breast cancer risk, particularly in populations with a high prevalence of this allele, such as Mexican women. Understanding the genetic predisposition can aid in the early identification of high-risk individuals and tailor prevention and treatment strategies accordingly. Moreover, targeting the VEGFA pathway could offer therapeutic benefits, especially in cases where angiogenesis plays a pivotal role in tumor progression [31]. In addition, according to others’ therapeutic strategies, studies such as the one by Damiano-Gentile have included patients carrying mutations in the *BRCA1* and *BRCA2* genes. In this study, the outcomes of patients treated with chemotherapy and breast preservation surgery were compared with those who underwent radical mastectomy. The results showed similar survival rates in both groups, which contrasts with the previous belief that complete breast removal was the best option [32].

In parallel, molecular markers like VEGFA have been investigated in therapies based on monoclonal antibodies that inhibit angiogenesis. A study by Gonzalez-Vacarreza identified that patients with the rs3025039 variant in the homozygous CC genotype responded better to these therapies compared to those with the CT or TT genotypes in metastatic colon cancer [31]. Furthermore, another study by P. Gampenrieder in metastatic breast cancer patients in Austria showed that those with the TT genotype who received chemotherapy treatment with bevacizumab had a shorter survival time compared to those with CT and CC genotypes [21]. These findings highlight the importance of continuing to investigate such markers in breast cancer patients, which could lead to more personalized and effective treatments.

While the study provides compelling evidence for the association between the rs3025039 variant and BC risk, it also highlights the need for further research to confirm these findings across diverse populations and larger sample sizes. Additionally, functional studies are necessary to elucidate the precise mechanisms by which the rs3025039 variant influences VEGFA expression and activity. Exploring the interaction between genetic factors and environmental or lifestyle influences would also provide a more comprehensive understanding of BC etiology.

Despite the controversial findings on the effect of the rs3025039 variant on BC, the present study clearly indicates a risk associated with the presence of the T allele and the CT genotype in BC patients. Although it has been previously suggested that the T allele is associated with decreased VEGFA protein levels and a consequently reduced risk of BC, we consider that other molecular mechanisms might be at play in VEGFA that could be contributing to the risk observed in our population. Hypothetically, the following could be occurring: (1) the rs3025039 variant may be in linkage disequilibrium with other variants in the same gene; (2) the rs3025039T variant might act as a mediator in thrombosis rather than angiogenesis; (3) perhaps VEGFA is more likely to affect tumor biological behaviors than define susceptibility to tumors; (4) therefore, the rs3025039T variant might be more effective as a predictive and prognosis marker rather than as a risk factor [33]; and finally, (5) it is known that 3′-UTR regions of mRNA contain regulatory regions of gene expression and regulatory proteins. By binding to these regions, miRNAs can increase or decrease the expression of various mRNAs by regulating translation directly.

Some recommendations and/or limitations of the study were as follows: (a) in the present study, the concentration of the VEGF protein and mRNA levels were not determined, so it was not possible to corroborate which patients produce more or less protein or mRNA according to their genotype. (b) There is a possible limitation in the statistical power of the study, so we consider it necessary to confirm the findings with more extensive genetic studies. (c) The results reported in the present study are specific to the study population (Mexican), so it is not recommended that they be extrapolated to other populations. (d) Ideally, it would be interesting, by means of a follow-up study, to evaluate the progression of breast cancer in those patients who have the T allele or CT genotype, since according to the results obtained, it would be thought that those patients carrying the T allele or CT genotype would have a greater possibility of progressing to a higher stage of BC due to the presence of angiogenesis.

## 4. Materials and Methods

### 4.1. Study Subjects

We conducted a transversal study, conformed by the BC group, which included 231 women with a clinical and histopathological confirmed diagnosis of sporadic BC, and were recruited as part of the “ELLA Binational Breast Cancer Study”, a multicenter study designed to look for molecular markers of breast tumors, clinicopathological characteristics, and risk factors in women of Mexican descent [34]. Their clinical features, including menopausal status, body mass index, molecular phenotype, hormonal receptors, clinical stadium, vital state, and metastasis, were obtained through medical records. Additionally, we included a reference group (RG), which was composed of 201 healthy women, who upon questioning, did not report having BC or evidence of infectious, heart-related, inflammatory, or renal diseases and were without a history of recent surgery or blood transfusions. All participants were over 18 years of age, born in the state of Jalisco, Mexico, with ethnic ancestry spanning three generations in western Mexico. They willingly agreed to participate and gave their informed written consent. This study was carried out with the approval number CI-9708 granted by the Ethics and Research Committee of the Universidad de Guadalajara.

### 4.2. Genotyping

A 7 mL sample of peripheral blood was collected in an EDTA-anticoagulated tube from both study groups. DNA was extracted from the leukocyte samples using the salting-out method of Miller et al., 1988 [35]. The extracted DNA was quantitated using the Nanodrop 2000 (Thermo Fisher Scientific, Waltham, MA, USA) and adjusted to a concentration of 100 ng/µL.

Molecular genotyping for rs3025039 was performed using PCR–RFLP (polymerase chain reaction–restriction fragment length polymorphism). The PCR reactions were performed using 10 ng of genomic DNA; the mix consisted of 1X PCR buffer, 2.0 mM MgCl_2_, 200 μM of each dNTP, 0.5 pmol of each primer, 0.05 U of Taq DNA polymerase (Life Technologies Corporation, Carlsbad, CA, USA), and water, all in a total volume of 10 µL. The primers used had the following sequence: forward: AAG GAA GAG GAG ACT CTG CG and reverse: TAA ATG TAT GTA TGT GGG TGG GT, which generated a 208 bp fragment [36]. The PCR conditions were an initial denaturation at 94 °C for 5 min, followed by 30 cycles of 94 °C, 59 °C, and 72 °C for 30 s each, and a final extension at 72 °C for 7 min. The reaction was carried out on a PTC-200 thermal cycler (MJ Research, Waltham, MA, USA).

A total of 5 µL of amplified product was digested with one unit of *Nla III* enzyme (NEB, New England Biolabs, Ipswich, MA, USA), 1X *Nla III* buffer, and 1X BSA. The final reaction of 10 µL was taken at 37 °C for 3 h on the same thermocycler. The presence of the T allele generated a cleavage site, producing fragments of 86 bp and 122 bp, while the presence of the C allele did not generate a cleavage site, leaving the 208 bp fragment intact. The amplified and digested fragments were visualized by polyacrylamide gel electrophoresis (29:1) at 6% using an OWL P9DS camera (Thermo Fisher Scientific, Waltham, MA, USA) and stained with silver nitrate (Golden Bell reagents, Jalisco, Mexico). Genotypes were determined by observing band size, which was compared to a 25 bp molecular weight marker (Invitrogen, Life Technologies Corporation, Carlsbad, CA, USA). To ensure quality control, water was used instead of DNA as a target, and positive samples for each allele were used as a positive control in the *Nla III* digestion. Additionally, 10% of all samples were repeated as an internal control to verify genotyping, and all results were confirmed by an independent blinded observer.

### 4.3. Static Analysis

Using frequencies and percentages, continuous and categorical variables were analyzed to determine their distributions. Allele frequencies were calculated by counting genotypes using IBM SPSS Statistics (SPSS Inc., Chicago, IL, USA, v25.0) by counting genotypes. The Hardy–Weinberg equilibrium (HWE) of the RG was calculated based on observed versus expected proportions using the method SNPStat (https://www.snpstats.net/, accessed on 27 March 2023). Both intergroup and intra-group analysis was performed using exact and χ^2^ tests.

## 5. Conclusions

The T allele and CT genotype are linked to increased BC risk, but this association may reflect tumor biological behaviors rather than a direct causal relationship. Furthermore, we consider it necessary to conduct more studies to confirm these findings and explore the underlying mechanisms.

## Figures and Tables

**Table 1:** Characteristics of BC patients.

Clinical Feature	*n* (%)
Menopause status	
Premenopause	71 (0.32)
Postmenopause	158 (0.68)
Molecular phenotype	
Luminal A	64 (0.38)
Luminal B	33 (0.20)
HER2	37 (0.22)
TNBC	33 (0.20)
Body mass index	
Underweight	1 (0.01)
Normal weight	64 (0.29)
Overweight	86 (0.39)
Obesity class I	52 (0.23)
Obesity class II	11 (0.05)
Obesity class III	7 (0.03)
Hormonal receptors	
ER-positive	143 (0.64)
ER-negative	82 (0.36)
PR-positive	125 (0.56)
PR-negative	97 (0.44)
HER2-positive	52 (0.27)
HER2-negative	142 (0.73)
Metastase status	
Without metastasis	174 (0.83)
With metastasis	35 (0.17)
Clinical stadium	
I	28 (0.14)
II	105 (0.52)
III	68 (0.34)

**Table 2:** Frequencies of alleles and genotypes, along with the results of the association test for the rs3025039 variant in the *VEGFA* gene.

Inheritance Model	Reference Group *n* (%)	Breast Cancer *n* (%)	*p*-Value	OR (95% CI)
Allele				
C	314 (0.78)	318 (0.69)	0.0038	Reference
T	88 (0.22)	144 (0.31)		1.61 (1.18–2.19)
Co-dominant model				
C/C	120 (0.59)	89 (0.39)	<0.0001 NS	Reference
C/T	74 (0.38)	140 (0.60)		2.55 (1.75–3.78)
T/T	7 (0.03)	2 (0.01)		
Dominant model				
C/C	118 (0.59)	89 (0.39)	<0.0001	Reference
C/T + T/T	83 (0.41)	142 (0.62)		2.36 (1.61–3.48)
Recessive model				
C/C + C/T	194 (0.97)	229 (0.99)		Reference
T/T Overdominant	7 (0.03)	2 (0.09)	0.053	0.24 (0.05–1.18)
C/C + T/T	127 (0.63)	91 (0.39)	<0.0001	Reference
C/T	74 (0.37)	140 (0.61)		2.64 (1.79–3.90)

OR: odds ratio; *p*: significance defined by the χ^2^ test; CI: confidence interval; NS: no significance.

**Table 3:** Association of rs3025039 variant genotypes of BC patients and their clinical features.

BC Patients							
*n* of Patients with Genotype rs3025039							
rs3025039	CC	CT	*p*-Value	OR (95% CI)	TT	*p*-Value	OR (95% CI)
Menopause status							
Premenopause	35	35		Reference	1		
Posmenopause	54	103	0.027	1.91 (1.08–3.38)	1	0.762	0.65 (0.04–10.70)
Molecular Phenotype							
Luminal A	25	38		Reference	1		
Luminal B	10	23	0.366	1.51 (0.62–3.71)	0	*	
HER2/neu	13	23	0.891	1.16 (0.50–2.72)	0	*	
TNBC	14	19	0.967	0.89 (0.38–2.10)	1	0.727	1.79 (0.10–30.81)
Vital state							
Live free of BC	55	84		Reference	0		
Live with BC	15	21	0.818	0.92 (0.44–1.93)	1	0.152	10.74 (0.42–276.98)
Metastasis							
Without metastases	67	106		Reference	1		
With metastases	14	20	0.789	0.90 (0.43–1.91)	1	0.278	4.79 (0.28–81.18)
Hormone receptors							
Estrogen receptor+	55	87		Reference	1		
Estrogen receptor−	32	49	0.909	0.97 (0.55–1.69)	1	0.705	1.72 (0.10–28.43)
PR^+^	54	70		Reference	1		
PR^−^	32	64	0.125	1.54 (0.89–2.68)	1	0.714	1.69 (0.10–27.92)
HER2^+^	19	32		Reference	1		
HER2^−^	56	85	0.757	0.90 (0.47–1.74)	1	0.453	0.34 (0.02–5.69)

This table presents the odds ratios (ORs) for individuals with the CC genotype (wild type) compared to those with heterozygous CT and homozygous mutated TT genotypes; *p*: significance defined by the χ^2^ test; CI: confidence interval; TNBC: triple-negative BC, *: values not calculated because no TT genotypes were observed.

## Data Availability

Research data are stored in our laboratory storage unit.
